# Discretionary foods and drinks in Norwegian children and adolescents’ diet: data from the national dietary survey Ungkost 3

**DOI:** 10.1017/S1368980023001982

**Published:** 2023-12

**Authors:** Mari Mohn Paulsen, Jannicke Borch Myhre, Torunn Holm Totland, Lene Frost Andersen

**Affiliations:** 1 Department of Nutrition, Institute of Basic Medical Sciences, University of Oslo, Post Box 1110 Blindern, Oslo, 0317, Norway; 2 Physical Health and Ageing, Norwegian Institute of Public Health, Oslo, 0213, Norway

**Keywords:** Discretionary foods, Sustainable diet, Dietary research, Children and adolescents

## Abstract

**Objective:**

This study aimed to identify the amount of discretionary foods and drinks consumed by Norwegian children and adolescents, describe how such products contribute to the intake of total energy and nutrients, and study the distribution in intake of discretionary foods and drinks across different meals. Secondly, the aim was to explore factors associated with those children and adolescents having the highest consumption of discretionary foods and drinks.

**Design:**

Secondary analysis of data from a national survey of dietary intake among Norwegian children and adolescents.

**Setting:**

Schools in fifty randomly selected municipalities in Norway.

**Participants:**

The study population included 636 pupils in 4^th^ grade (9–11 years) and 687 pupils in 8^th^ grade (12–14 years).

**Results:**

Discretionary foods and drinks contributed to about 20 % of the children and adolescents’ total energy intake. These products contributed to about two-thirds of the participants’ intake of added sugar, and limited amounts of dietary fibre, vitamins and minerals. The quartile which had the lowest proportion of their energy intake from discretionary foods and drinks seemed to have a higher intake of whole grains, and fish and seafood.

**Conclusions:**

Almost all 4^th^ and 8^th^ graders in Norway consumed discretionary foods and drinks, and these products contributed to a substantial proportion of the total energy intake and limited amounts of nutrients. Those children and adolescents consuming the least discretionary foods and drinks had a higher intake of whole grains, fish and seafood, indicating healthier and more sustainable food habits.

Tackling the overconsumption of discretionary foods has been described as important for both human and planetary health^(1)^. Discretionary foods are defined by the Australian Dietary Guidelines^(2)^ as ‘…foods and drinks not necessary to provide nutrients the body needs, but that may add variety’. Such foods and drinks are often high in energy, saturated fat, added sugar, salt or alcohol. Foods and drinks typically included in this definition are sugar-sweetened drinks, cakes, chocolate, ice cream and salty snacks. Non-discretionary foods are contrary, food and beverage items belonging to the core food groups: cereals, vegetables, fruits, legumes, nuts and seeds, fresh meat and fish, and dairy^(1)^.

From a health perspective, excessive consumption of energy-dense and nutrient-poor food items is associated with an increased risk of CHD, diabetes type 2, some cancer types and dental caries^(3,4)^. From a sustainability point of view, the environmental impact of discretionary food products is suggested to be substantial, due to the high degree of processing and packaging related to these products^(5)^. The EAT-Lancet Commission described the healthy sustainable reference diet in 2019^(6)^. This diet included plenty of vegetables, fruits, whole grains, legumes, nuts and unsaturated oils. It included, however, a low quantity of foods and components which are typically prevalent in discretionary foods, such as red meat, processed meat, added sugar, refined grains and starchy vegetables^(6)^. In Australia and New Zealand, discretionary foods are estimated to contribute about 30 % of the total food-related greenhouse gas emissions (GHGe)^(1,7–9)^. It is argued that reducing the production and consumption of discretionary food products should be seen as a crucial step towards aligning human and planetary health and creating a more sustainable food system^(1,10)^.

In Denmark, new guidelines for the maximum intake of discretionary foods and drinks were recently suggested for both children and adults, due to both nutritional and sustainability aspects^(11)^. In Norway, the food-based dietary guidelines recommend maintaining a good balance between the amount of energy consumed and the amount of energy spent through physical activity and limiting the consumption of energy-dense foods and drinks that are high in sugar, saturated fat and salt^(12)^. However, we do not have specific guidelines for discretionary foods, and little is known about the amount consumed among children and adolescents and how such foods and drinks affect the quality of their diet from a nutritional perspective.

The present study aimed to identify the amount of discretionary foods and drinks consumed by Norwegian children and adolescents, describe how such products contribute to the intake of total energy and nutrients, and study the distribution in intake of discretionary foods and drinks across different meals. Secondly, we aimed to explore factors associated with children and adolescents having the highest consumption of discretionary foods and drinks.

## Methods

### Study population and design

**Fig. 1:**
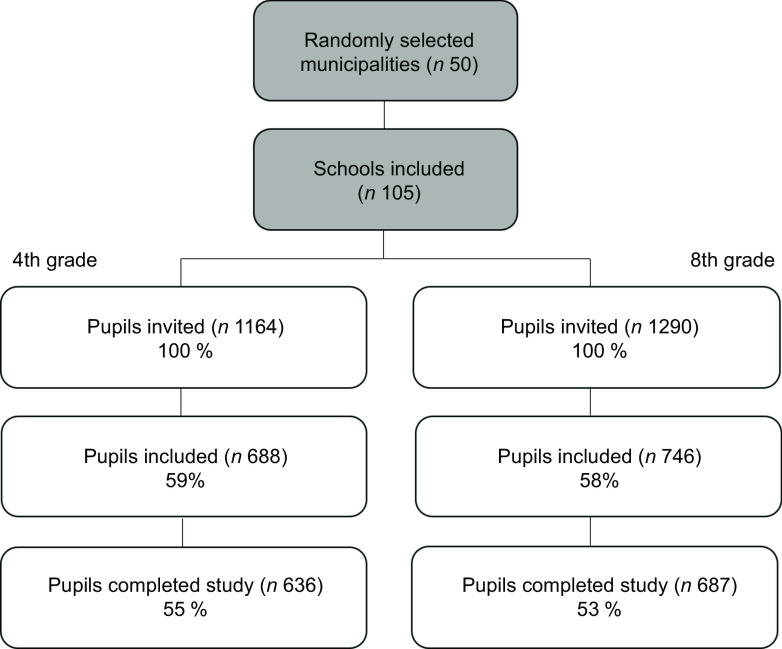
Flow diagram of recruitment and participation

This study was a secondary analysis of data from the ‘Ungkost 3’-study, which is a national survey of dietary intake among Norwegian children in 4^th^ grade (9–11 years) and 8^th^ grade (12–14 years)^(13)^. The data were collected during the second half of 2015 and included 108 **schools** in fifty randomly selected municipalities in Norway. The final sample included 636 pupils in 4^th^ grade and 687 pupils in 8^th^ grade, representing a participation rate of 55 % and 53 % respectively, as illustrated in Fig. 1. All invited pupils were given information about the study and their parents or guardians signed a written consent form. The pupils were instructed through a physical classroom demonstration on how to record their dietary intake in a web-based food diary (WebFR) for 4 d. The start of the registration period was either a Wednesday or a Sunday to secure that 3 weekdays (Monday – Friday), and 1 weekend day (Saturday – Sunday) were included for all participants.

### Assessment of dietary intake

The WebFR was based on the Danish Web-based Dietary Assessment Software for Children (WebDASC)^(14)^ validated for use among 9- to 11-year-old Danish children^(15)^ and later validated in a similar Norwegian population^(16,17)^. The food diary was developed and adapted for use in a Norwegian context including a database with 570 of the most commonly used food and beverage items in Norway. Pictures showing portion sizes in a series of two to four pictures were included. The food diary was structured around six meals including three main meals (breakfast, lunch and dinner) and three snacks in between. An interactive character was used to guide the participants through the day’s eating occasions. As an evening meal is common in Norway, the snack after dinner was defined as ‘supper’ for the analyses included in this paper. The remaining two snacks between breakfast and lunch, and lunch and dinner, were merged into a common ‘snack’. The food and beverage items could be recorded through drop-down lists with different categories or through free text search. The recording included portion size estimations and the number of portions consumed. The food diary included prompting questions regarding the use of butter or margarine, bread spreads and beverages with the meal. At the end of the day, the participant was shown an overview of all recorded food and beverage items during the day with the possibility of adding or deleting food items. The food diary was designed in such a way that the children and adolescents could do a lot of the recording themselves in collaboration with their guardians. Guardians were instructed to assist the youngest participants (4^th^ graders). After completing each day’s recording, the participant got access to a computer game as a reward. If the participant forgot to record their dietary intake, an automated email was sent to the participants’ guardians as a reminder.

### Participant characteristics

Information about the children’s and adolescents’ age, sex, self-reported weight and height and parental education was provided from questions included in the written consent form. Parental education level was categorized into low and high education. A low education level was defined as both parents and guardians having a high school education or lower whereas a high education level was defined as at least one parent or guardian having a university or university college degree. Underweight, normal weight, overweight and obese participants were defined by using the age and sex-specific BMI (ISO-BMI) defined by Cole and Lobstein^(18)^. Due to few obese participants, overweight and obesity were merged into one category when comparing quartiles.

### Definition of discretionary foods and drinks

In the present study, we included cakes, buns, waffles, muffins, biscuits and other pastries, potato crisps, chips and similar snacks, ice cream, including sorbet, sweet desserts, all sugar-sweetened drinks including soft drinks, fruit drinks, sports drinks and energy drinks, sugar confectionaries, including chocolate, sugar candy or sweets, sweet spreads and sweet snack bars in the definition of discretionary foods. This definition was based partly on previous work by Medin *et al.* in the same study population^(19)^ and recent studies in another Norwegian^(20)^, a Danish^(21)^ and seven European populations^(22)^.

### Estimated energy requirements

The estimation of average energy requirements was based on the Nordic Nutrition Recommendations reference values for the age groups and an average physical activity level^(10)^. For the 4^th^ graders, the estimated daily energy requirement was 7120 kJ for girls and 7660 kJ for boys using reference values for 9-year-olds with an average physical activity level of 1·57. The corresponding values for the 8^th^ graders were 9170 kJ for the girls and 9990 kJ for the boys using an average physical activity level of 1·73^(10)^.

### Data analysis and statistics

The dietary calculation programme ‘Kostberegningssystem’, database version AE-14, at the University of Oslo, Norway^(23)^ was used to calculate the dietary variables. Descriptive analyses were conducted to assess the background characteristics of the participants, to calculate the participants’ intake of discretionary foods and drinks, their contribution of nutrients and their distribution across meals. For micronutrients, analyses were conducted for Vitamin D, calcium and iron as these nutrients previously have been shown to be below recommended intakes in groups of this population and they are particularly important for children and adolescents who are still growing. Dietary supplements were excluded from the analyses of dietary intake.

To test differences between participants in the lowest quartile (≤25) and the highest quartile (≥75) of discretionary food and drink intake concerning background variables, the Pearson chi-squared test or Ficher’s exact test was used. For selected food groups, the Independent samples *t* test was used. As the results revealed significant differences between boys and girls, the analyses were done separately for both genders.

All statistical analyses were conducted in IBM SPSS Statistics version 28. The tests conducted used a two-sided significance level of 1 %, to account for multiple testing.

### Ethics

The study was acknowledged by the Norwegian Centre for Research Data (reference number: 41 946). Written consent was obtained from the parents/guardians of all participants. All children and adolescents completing the study received a gift certificate worth 200 NOK (approximately 20 EUR).

## Results

### Characteristics of the study population

The background characteristics of the participants in Ungkost 3 are shown in Table 1. The age ranged from 8 to 15 years, with the majority of 4^th^ graders being 9 years old and the majority of 8^th^ graders being 13 years old. Most of the children in both 4^th^ and 8^th^ grade (77 %) were of normal weight, and 82 % of the 4^th^ graders and 71 % of the 8^th^ graders came from families where at least one of the parents/guardians had education from a university or university college.

**Table 1:** Background characteristics of the study population in Ungkost 3

Characteristics	4^th^ graders (*n* 636)		8^th^ graders (*n* 687)	
	*n*	%	*n*	%
Sex				
Boy	295	46·4	332	48·3
Girl	341	53·6	355	51·7
	Mean	sd	Mean	sd
Age (years)	9	0·4	13	0·5
	*n*	%	*n*	%
BMI category*,†				
Underweight	37	6·2	46	7·1
Normal weight	458	76·7	498	77·1
Overweight and obese	102	16·0	102	13·5
Parental educational level‡,§				
Low	114	18·3	201	29·4
High	509	81·7	482	70·6

* Based on Cole and Lobstein, using ISO-BMI^(18)^.

† Missing BMI values: *n* 39 for 4^th^ graders and *n* 41 for 8^th^ graders.

‡ Low education is defined as both parents/guardians having an education maximum at the high school level. High education is defined as at least one parent/guardian having a university or university college degree.

§ Missing parental education values: *n* 13 for 4^th^ graders and *n* 4 for 8^th^ graders.

### Intake of discretionary foods

The majority of the children and adolescents included some types of discretionary foods and drinks in their diet, as shown in Table 2. Discretionary foods and drinks constituted about 20 % of the total energy intake for both the 4^th^ and the 8^th^ graders, and also related to estimated reference energy requirements as defined by the Nordic Nutrition Recommendations^(10)^. Sugars-sweetened drinks constituted about 60 % of the total intake of discretionary foods and drinks in grams in both age groups.

**Table 2:** Intake of discretionary foods and drinks (DF) among 4^th^ graders and 8^th^ graders

	4^th^ graders (*n* 636)		Consumers	8^th^ graders (*n* 687)		Consumers
	Mean	sd	%	Mean	sd	%
Mean daily intake of DF*, g	235	179	97·3	300	299	96·5
Sugar-sweetened drinks†	133	143	76·3	193	253	77·9
Cakes, biscuits, buns‡	37	43	73·1	36	51	65·1
Desserts§	22	56	24·7	17	47	18·9
Sugar confectionaries‖	21	26	68·6	29	46	68·7
Ice cream and sorbet	9	18	31·3	8	22	23·6
Sweet spreads¶	4	11	26·3	6	17	26·8
Potato crisps, chips and similar snacks	8	15	46·5	11	18	48·5
Mean daily energy intake from DF*, kJ	1478	969		1784	1524	
DF* proportion of total energy intake (kJ), %	19·8	11·3		20·8	13·2	
DF* proportion of reference energy requirement**, %	20·2	13·2		18·6	16·0	

* DF: Discretionary foods.

† Includes soft drinks, cordial and iced tea.

‡ Includes cakes, muffins, waffles, buns, biscuits and other pastries.

§ Includes puddings, jelly, whipped cream, etc.

‖ Includes chocolate, sugar candy and sweets.

¶ Including chocolate spread, honey and other sweet spreads.

** Based on estimated daily energy requirements for children and adolescents in Nordic Nutrition Recommendations 2012^(10)^.

**Fig. 2:**
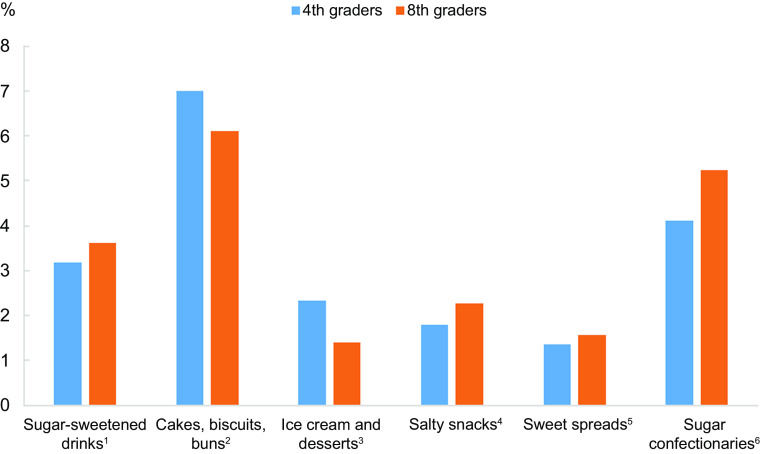
Percentage contribution of discretionary foods and drinks to the total energy intake among the 4^th^ and 8^th^ graders. Non-discretionary foods and drinks are not included in the figure. ^1^Includes soft drinks, cordial and iced tea. ^2^Includes cakes, muffins, waffles, buns, biscuits and other pastries. ^3^Includes ice cream, sorbet, puddings, jelly, whipped cream, etc. ^4^Includes crisps, chips and similar snacks. ^5^Includes chocolate spread, honey and other sweet spreads. ^6^Includes chocolate, sugar candy and sweets

Figure 2 illustrates how different types of discretionary foods and drinks contributed to the total energy intake among the 4^th^ and 8^th^ graders. Cakes, biscuits and buns constituted the largest contribution of the total energy intake in both age groups, followed by sugar confectionaries and sugar-sweetened drinks.

The contribution of selected nutrients from discretionary foods and drinks is shown in Table 3. Discretionary foods and drinks constituted about two-thirds of the total daily intake of added sugar for both the 4^th^ and the 8^th^ graders. About 17 % of the total intake of saturated fat and about 8 % of the total salt intake came from discretionary foods and drinks. Discretionary foods and drinks contributed to about 10 % of the daily intake of dietary fibre and calcium, about 8 % of the protein intake, and about 10 % of the daily intake of vitamin D and iron in both age groups.

**Table 3:** Mean daily intake of nutrients* from discretionary foods and drinks (DF) and the total diet

	4^th^ graders (*n* 636)						8^th^ graders (*n* 687)					
	From DF†		Total intake		DF† proportion, % of total intake		From DF†		Total intake		DF† proportion, % of total intake	
	Mean	sd	Mean	sd	Mean	sd	Mean	sd	Mean	sd	Mean	sd
Added sugar, g	37	27	52	30	64·8	23·8	47	47	62	51	68·6	25·0
Protein, g	5	4	68	21	8·3	6·4	6	6	75	26	8·0	7·0
Saturated fat, g	4·6	3·8	26·0	10·0	17·3	12·2	5·4	5·5	29	12	17·5	13·9
Salt, g	0·4	0·4	5·8	2·0	8·1	7·6	0·5	0·5	6·3	2·3	7·9	7·8
Dietary fibre, g	1·5	1·4	16	6	9·4	8·7	1·7	1·7	16·0	6·0	10·9	10·4
Vitamin D, µg	0·5	0·6	3·8	2·7	14·5	17·2	0·4	0·6	3·9	3·4	10·2	16·3
Calcium, mg	71	63	807	326	9·7	8·6	80	82	832	392	10·3	10·3
Iron, mg	0·7	0·6	8·0	2·0	9·7	8·0	0·9	0·8	8·0	3·0	10·2	8·8

* Dietary supplements are excluded from the analysis.

† DF: Discretionary foods and drinks.

**Fig. 3:**
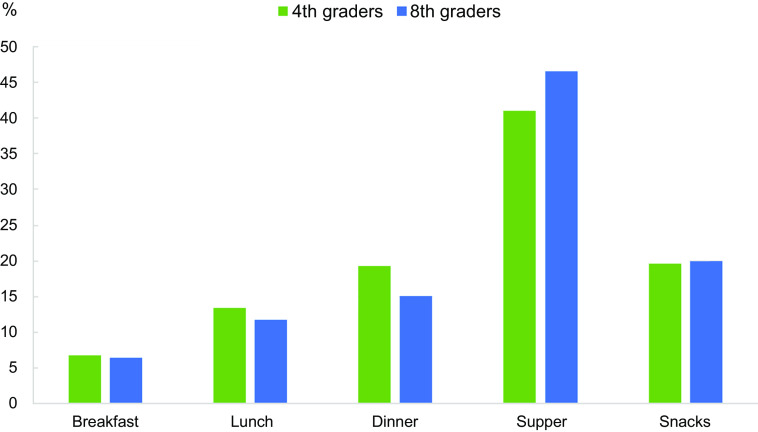
Percentage distribution for the intake of energy (kJ) from discretionary foods and drinks at different meals among the 4^th^ and 8^th^ graders

The supper meal contributed more than 40 % and the snacks contributed about one-fifth of the total energy from discretionary foods and drinks in both age groups (Fig. 3). The breakfast and lunch meals contributed the least to the total discretionary foods and drinks consumption.

A wide distribution in the intake of discretionary foods and drinks was found among children and adolescents. Almost 60 per cent of the 4^th^ and 8^th^ graders in the quartile having the highest (≥75 percentile) proportion of their energy intake from discretionary foods and drinks were girls. Due to significantly more girls than boys in the highest quartile, the analyses for other background characteristics were done separately for each gender, as shown in Tables 4 and 5.

**Table 4:** Characteristics among 4^th^ graders in the lowest and highest quartile for the proportion of daily energy intake from discretionary foods and drinks

	Boys				*P*-value*	Girls				*P*-value*
	≤25 (*n* 84)		≥75 (*n* 66)			≤25 (*n* 75)		≥75 (*n* 94)		
	Mean	sd	Mean	sd		Mean	sd	Mean	sd	
Mean energy from DF†, %	6·9	3·6	35·5	8·1		6·7	3·7	34·7	6·9	
	*n*	%	*n*	%		*n*	%	*n*	%	
BMI category‡,§										
Underweight or normal weight	68	84·0	53	84·1	0·977	56	82·4	68	78·2	0·517
Overweight or obese	13	16·0	10	15·9		12	17·6	19	21·8	
Parental education‖,¶										
Low	18	22·5	16	24·6	0·765	16	21·9	19	20·7	0·843
High	62	77·5	49	75·4		57	78·1	73	79·3	
Speartime sports activity**,††										
≤ 1 h	14	19·2	14	22·2	0·590	7	10·4	23	25·8	0·003
2–3 h	26	35·6	26	41·3		32	47·8	49	55·1	
≥ 4 h	33	45·2	23	36·5		28	41·8	17	19·1	
Walking or bicycling to school‡‡										
Never (use car or public transportation)	23	31·5	14	22·2	0·018	13	19·7	16	18·0	0·029
Sometimes (1–4 times/week)	14	19·2	4	6·3		4	6·1	19	21·3	
Everyday (5 times a week)	36	49·3	45	71·4		49	74·2	54	60·7	
TV/DVD weekdays§§					0·420					0·320
≤ 1/2 h	24	32·9	22	35·5		22	33·3	20	22·7	
1/2–1 h	41	56·2	29	46·8		35	53·0	52	59·1	
≥ 2 h	8	11·0	11	17·7		9	13·6	16	18·2	
TV/DVD weekend days‖‖					0·106					0·815
≤ 1/2 h	5	6·9	9	14·3		4	6·2	4	4·5	
1/2–1 h	32	44·4	18	28·6		21	32·3	26	29·2	
≥ 2 h	35	48·6	36	57·1		40	61·5	59	66·3	
Gaming TV/computer weekdays¶¶					0·149					0·095
≤ 1/2 h	48	68·6	31	51·7		58	95·1	72	84·7	
1/2–1 h	19	27·1	25	41·7		2	3·3	11	12·9	
≥ 2 h	3	4·3	4	6·7		1	1·6	2	2·4	
Gaming TV/computer weekend days***					0·105					0·854
≤ 1/2 h	41	56·9	24	38·7		52	83·9	69	80·2	
1/2–1 h	19	26·4	22	35·5		6	9·7	10	11·6	
≥ 2 h	12	16·7	16	25·8		4	6·5	7	8·1	

* Chi-square of Fishers’ exact test. Fisher’s exact test for Girls TV weekend days and Gaming weekend days, and for girls and boys Gaming weekdays. Significance level *P* < 0·01.

† DF: Discretionary foods and drinks.

‡ Based on Cole and Lobstein^(18)^.

§ Missing BMI values *n* 20.

‖ Low education: both parents/guardians have an education maximum at the high school level. High education: at least one parent/guardian having a university or university college degree.

¶ Missing parental education values: *n* 9.

** Number of h/week of sports activities outside school hours.

†† Missing sports activities values: *n* 27.

‡‡ Missing walking or bicycling to school values: *n* 28.

§§ Missing TV/DVD weekdays: *n* 30.

‖‖ Missing TV/DVD weekend days: *n* 30.

¶¶ Missing gaming weekdays: *n* 43.

*** Missing gaming weekend days: *n* 37.

**Table 5:** Characteristics among 8^th^ graders in the lowest and highest quartile for the proportion of daily energy intake from discretionary foods and drinks

	8^th^ graders									*P*-value*
	Boys				*P*-value*	Girls				
	≤25 (*n* 95)		≥75 (*n* 76)			≤25 (*n* 76)		≥75 (*n* 96)		
	Mean	sd	Mean	sd		Mean	sd	Mean	sd	
Mean energy from DF†, %	5·8	3·3	39·7	8·8		5·3	3·3	38·0	8·1	
	*n*	%	*n*	%		*n*	%	*n*	%	
BMI category‡,§										
Underweight	6	6·6	6	8·6	0·881	5	6·8	5	5·6	0·962
Normal weight	70	76·9	52	74·3		54	73·0	67	74·4	
Overweight or obese	15	16·5	12	17·1		15	20·3	18	20·0	
Parental education‖,¶										
Low	27	28·4	26	34·7	0·383	20	26·7	27	28·4	0·800
High	68	71·6	49	65·3		55	73·3	68	71·6	
Speartime sports activity**,††										
≤ 1/2 h	7	9·0	16	22·2	0·076	7	11·5	7	8·0	0·492
1–3 h	26	33·3	22	30·6		17	27·9	32	36·4	
≥ 4 h	45	57·7	34	47·2		37	60·6	49	55·7	
Walking or bicycling to school‡‡										
Never (use car or public transportation)	16	20·5	27	37·5	0·058	21	35	31	35·2	0·286
Sometimes (1–4 times/week)	7	9·0	7	9·7		3	5	11	12·5	
Everyday (5 times a week)	55	70·5	38	52·8		36	60	46	52·3	
TV/DVD weekdays§§										
≤ 1/2 h	32	42·1	25	34·7	0·017	27	44·3	26	29·5	0·180
1/2–1 h	36	47·4	26	36·1		23	37·7	43	48·9	
≥ 2 h	8	10·5	21	29·1		11	18·0	19	21·6	
TV/DVD weekend days‖‖					0·337					0·196
≤ 1/2 h	17	22·4	16	22·2		16	26·2	13	15·1	
1/2–1 h	28	36·8	19	26·4		18	29·5	34	39·5	
≥ 2 h	31	40·8	37	51·4		27	44·3	39	45·3	
Gaming TV/computer weekdays¶¶					0·054					
≤ 1/2 h	24	32·0	28	38·9		57	93·4	71	83·7	0·203
1/2–1 h	28	37·3	14	19·4		2	3·3	8	9·3	
≥ 2 h	23	30·7	30	41·7		2	3·3	6	7·0	
Gaming TV/computer weekend days***					0·523					0·376
≤ 1/2 h	14	18·4	16	22·2		54	88·5	69	80·2	
1/2–1 h	23	30·3	16	22·2		3	4·9	9	10·5	
≥ 2 h	39	51·3	40	55·6		4	6·6	8	9·3	

* Chi-square test. Fisher’s exact test for girls Gaming weekdays and Gaming weekend days. Significance level *P* < 0·01.

† DF: Discretionary foods and drinks.

‡ Based on Cole and Lobstein^(18)^.

§ Missing BMI values *n* 18.

‖ Low education: both parents/guardians have an education maximum at the high school level. High education: at least one parent/guardian having a university or university college degree.

¶ Missing parental education values: *n* 3.

** Number of h/week of sports activities outside school hours.

†† Missing sports activities values: *n* 44.

‡‡ Missing walking or bicycling to school values: *n* 45.

§§ Missing TV/DVD weekdays: *n* 46.

‖‖ Missing TV/DVD weekend days: *n* 48.

¶¶ Missing gaming weekdays: *n* 49.

*** Missing gaming weekend days: *n* 48.

Among the 4^th^ graders, a significantly higher proportion of the girls in the ≤25 percentile for discretionary food and drink intake had ≥4 h of sports activities outside school hours compared with those in the ≥75 percentile. However, this was not found for the boys in 4^th^ grade (Table 4) nor the 8^th^ graders (Table 5). No significant differences in discretionary food and drink intake were seen according to BMI category, parental education level or screen time (Tables 4 and 5).

Tables 6 and 7 show the intake of selected food groups among the 4^th^ and 8^th^ graders in the lowest and highest quartile for the proportion of daily energy intake from discretionary foods. Among the 4^th^ graders, the intake of fruit and vegetables, whole grain and fish and seafood was significantly higher for the children in the ≤25 percentile compared to the ≥75 percentile for discretionary food and drink intake. For the adolescents (8^th^ graders), the same tendency was found for whole grain and fish and seafood (Table 7). For red and processed meat, the opposite tendency was observed with a higher intake among those in the lowest quartile for discretionary foods compared to the highest. However, this was only significant for the girls in the 4^th^ grade. Among the 8^th^ graders, the energy intake was significantly higher among those in the ≥75 percentile for discretionary food and drink intake compared to the ≤25 percentile; however, this difference was not seen for the 4^th^ graders.

**Table 6:** Intake of selected food groups among 4^th^ graders in the lowest and highest quartile for the proportion of daily energy intake from discretionary foods and drinks

	4^th^ graders									*P*-value*
	Boys				*P*-value*	Girls				
	≤25 (*n* 84)		≥75 (*n* 66)			≤25 (*n* 75)		≥75 (*n* 94)		
	Mean	SD	Mean	SD		Mean	SD	Mean	SD	
Energy intake, kJ	7256	1741	7357	2165	0·753	7269	2084	7341	2114	0·825
Fruit and vegetables, g	167	111	112	94	0·002	168	88	128	80	0·002
Fruit, g	72	67	49	69	0·044	81	67	63	60	0·076
Vegetables, g	95	76	63	51	0·004	87	53	65	54	0·007
Read and processed meat, g	146	112	139	139	0·719	149	106	105	72	0·002
Red meat, g	97	74	91	75	0·633	97	62	70	46	0·002
Processed meat, g	50	45	48	68	0·871	52	50	35	35	0·011
Whole grain, g	60	33	46	33	0·011	55	34	37	20	<0·001
Fish and seafood, g	34	37	21	26	0·017	28	35	14	21	0·002

* Independent samples *t* test.

**Table 7:** Intake of selected food groups among 8^th^ graders in the lowest and highest quartile for the proportion of daily energy intake from discretionary foods and drinks

	8^th^ graders									*P*-value*
	Boys				*P*-value*	Girls				
	≤25 (*n* 95)		≥75 (*n* 76)			≤25 (*n* 76)		≥75 (*n* 96)		
	Mean	sd	Mean	sd		Mean	sd	Mean	sd	
Energy intake, kJ	7625	2664	9511	2915	<0·001	6097	2356	8796	2854	<0·001
Fruit and vegetables, g	140	112	112	94	0·079	150	99	135	108	0·343
Fruit, g	40	64	30	47	0·231	58	66	51	58	0·418
Vegetables, g	100	91	82	80	0·182	92	65	84	75	0·490
Read and processed meat, g	176	101	145	99	0·052	111	77	129	89	0·180
Red meat, g	129	81	102	65	0·019	83	58	96	70	0·226
Processed meat, g	46	42	43	45	0·636	28	30	33	32	0·274
Whole grain, g	60	34	40	25	<0·001	45	24	36	21	0·006
Fish and seafood, g	34	49	18	36	0·018	29	36	13	21	<0·001

* Independent samples *t* test.

## Discussion

The present study found that discretionary foods and drinks were consumed by almost all 4^th^ and 8^th^ graders in Norway and constituted more than 20 % of their total energy intake. Discretionary foods and drinks contributed to about two-thirds of the total daily intake of added sugar in both age groups and contributed with limited amounts of dietary fibre, vitamin D, calcium and iron. The supper meal contributed the most to the energy intake from discretionary foods and drinks in both age groups. There were significantly more girls than boys in the quartile with the highest proportion of energy intake from discretionary foods and drinks. The intake of whole grains, and fish and seafood was higher among the quartile of 4^th^ and 8^th^ graders having the lowest proportion of their energy intake from discretionary foods and drinks. The same tendency was seen for fruit and vegetables, but only for the 4^th^ graders.

### Definition of discretionary foods and drinks

The definition of discretionary foods and drinks varies between studies and has consequences for the estimates of total consumption. Studies conducted in Australia and New Zealand include processed meats, sausages, commercial burgers and pizza in the definition^(1,7–9)^, whereas studies from European populations,^(21,22)^ which are more comparable to a Norwegian setting, do not include such food items. In the present study, we included the definition as described in Denmark^(21)^ and other European populations^(22)^ and from a previous study using data from the same population^(19)^, which means that we did not include processed meats, sausages, burgers and pizza.

### Intake of discretionary foods and drinks

The observed contribution from discretionary foods and drinks to total energy intake in the present study corresponds with data from a recent Australian publication by Fayet-Moore *et al.* ^(24)^ using data from a nationally representative sample of almost 3000 children and adolescents from 2–18 years. Fayet-Moore *et al*. found that 99% of Australian children and adolescents consumed discretionary foods, in comparison to 97 % in the present study. In the present study, discretionary food and drinks constituted more than 20 % of the participants’ total energy intake. Even higher intake was found in the Australian study where more than one-third of daily energy intake came from discretionary foods and drinks for all age groups except children aged 2–3 years^(24)^, although as already explained, the definition of discretionary foods included more food groups in the Australian study.

According to Danish dietary recommendations, the consumption of discretionary foods and drinks should not exceed 5 % of the total energy intake among 9-year-olds and 6 % among 13-year-olds^(11)^. Denmark is a country comparable to Norway in several manners. The present study indicates that Norwegian children and adolescents consume about four times the maximum recommended amount, according to the Danish recommendations^(11)^.

Discretionary foods and drinks constituted more than two-thirds of the total daily intake of added sugar for both the 4^th^ and the 8^th^ graders and this exceeded the maximum recommended intake of 10 percentage of energy (E %) from added sugar. At the same time, discretionary foods and drinks contributed a very limited amount of dietary fibre, vitamins and minerals, strengthening the current definitions of discretionary foods and drinks as being of low nutritional quality^(1)^.

### Types of discretionary foods and drinks consumed and distribution between meals

Among the Norwegian children and adolescents, the discretionary food and beverage items contributing the most to the total energy intake were cakes, biscuits and buns, followed by sugar confectionaries and sugar-sweetened beverages in both age groups. Cakes, muffins, scones, cake-type desserts, sweet biscuits and pastries were the food items contributing the most to daily discretionary foods energy intake in the Australian study by Fayet-Moore *et al*.^(24)^. The present study indicated that the supper meal contributed the most to the energy intake of discretionary foods, followed by snacks. Fayet-Moore *et al*.^(24)^ found dinner to be the highest contributing meal, in addition to snacks.

As the food diary used in the present study was organized into six eating occasions including three main meals (breakfast, lunch and dinner) and three snacks, everything consumed after dinner was recorded in the last snack which was defined as supper, as described in the method section. This implies that both traditional Norwegian supper consisting of, for example, bread and milk and also typical snack products like potato crisps or sugar confectionaries are included in this meal category. Norway also has a tradition of children consuming sweets and other snacks particularly on Saturday evenings, which probably also has contributed to the higher intake of discretionary foods from supper.

### Characteristics of low *v*. high consumers of discretionary foods and drinks

We found significantly more girls than boys in the quartile with the highest contribution from discretionary foods and drinks to the total energy intake. We also found a tendency among the 4^th^ graders to a higher frequency of sports activities outside school hours among those girls having the lowest proportion of their energy intake from discretionary foods and drinks. It has previously been demonstrated that children and adolescents with higher levels of physical activity have a more preferable dietary pattern than those with lower physical activity levels^(25–28)^.

No significant differences in intake of discretionary foods were seen related to BMI, parental education level or screen time. The sample in the Ungkost study had parents/guardians with higher education compared to the general population in Norway^(29)^, and this may have affected the results. The Australian publication described above found no significant differences between sex, socio-economic status, physical activity or BMI. However, more females than males exceeded the recommended maximum discretionary foods and beverages servings, as females have lower maximum discretionary foods and beverages serving target due to lower mean energy requirements^(24)^.

### Implications for sustainability aspects

To add variation to the diet and to ensure a realistic dietary pattern, it is relevant to include some energy from discretionary foods and drinks^(30)^. However, the results from the present study show that the intake among Norwegian children and adolescents largely exceeds recommended maximum intake in comparable countries^(11)^.

The results in the present study indicated that the 4^th^ graders with the lowest proportion of daily energy intake from discretionary foods and drinks had higher intakes of fruit and vegetables, whole grains, and fish and seafood compared to those with the highest discretionary food and drink intake. The same tendency was found for whole grains and fish and seafood among the 8^th^ graders. Fruits and vegetables, whole grains and fish and seafood are food groups the population are recommended to increase the intake of, and which should be included in a sustainable diet^(6)^. The intake of red and processed meat was also higher among those 4^th^ graders with the lowest proportion of daily energy intake from discretionary foods and drinks, but only for the girls. A high intake of red and processed meat does not align with recommendations for a sustainable diet^(6,31)^. One could argue that the result with a higher intake of the food groups included in the present study in the ≤25 percentile for discretionary food intake implies that a low intake of discretionary food leads to a higher intake of whole foods at the expense of more processed foods, which typically characterize discretionary foods.

A recent systematic review of methods for combining health and environmental assessment of foods and diets argues that future research on sustainable diets would benefit from a greater focus on dietary changes that enable reduced intake of discretionary foods and excessive energy intake^(32)^. The Australian study by Hadjikakou^(1)^ showed that discretionary foods and drinks account for a significant part of the diet-related life cycle water use, GHGe and land use. A recent Danish study highlighted the need to avoid excessive consumption of discretionary foods and drinks to meet nutrient and food group recommendations without exceeding energy needs^(21)^. The authors argue that one of the main points to consider when adopting a more sustainable healthy plant-based diet is to limit discretionary foods and drinks^(21)^. This also complies with Australian data, showing a large potential to save dietary emissions by significantly reducing discretionary food intake^(9)^. A study among Swedish adults studying secular trends in diet-related GHGe since the year 2000 showed that men and women in all age groups decreased their dietary GHGe over the last 20 years and at the same time decreased their intake of discretionary foods^(33)^.

### National dietary guidelines

Several countries have included specific guidelines for the consumption of discretionary foods and drinks, such as Australia^(2)^, Denmark^(11)^ and the United States^(34)^. As such, food and beverage items contribute a high proportion of the intake of added sugar and limited amounts of nutrients, one could argue that Norway also should include guidelines for discretionary foods intake. As the environmental impact of discretionary food consumption is suggested to be substantial^(5)^, the recent work with revising the Nordic Nutrition Recommendations and the subsequent Norwegian revised recommendations should take these aspects into account.

A qualitative study among Australian children and adolescents suggested that the maintained excessive intake of discretionary foods and drinks may be partly due to misinterpretation of the national guidelines among the youth. Whilst Australian children and adolescents agreed that discretionary foods and drinks should be consumed only sometimes and in small amounts, they still interpreted this to represent regular intake^(35)^. A recent qualitative study in a Norwegian setting found that adolescents experience several barriers to healthy dietary behaviours and that these barriers varied with socio-economic position^(36)^. Among the adolescents in the lower socio-economic position neighbourhoods, fast food restaurants were a preferred social arena and western dishes were preferred over the food served at home^(36)^. In the current study, the parental education level was high which indicates a high socio-economic position among the participating children and adolescents.

### Strength and weaknesses

A strength of this study is that it includes a large, nationally representative sample size. Moreover, the WebFR has previously been validated in a similar sample as the current study sample using objective markers^(16,37)^. The WebFR was able to rank participants according to their intake of foods rich in carotenoids^(37)^; however, energy intake was significantly underestimated compared with total energy expenditure^(16)^. The data collection period did not include the spring and summer semester, and this may have influenced the results as typical seasonal products as, for example, strawberries or ice cream have been underestimated.

The participation rate in the present study was 55 % among the 4^th^ graders and 53 % among the 8^th^ graders. The high proportion of non-participating children and adolescents may affect the generalizability of the results. A large proportion of the children lived in families with one or more parents/guardians with high education level. In 2015, 39 % of individuals between 30–66 years in Norway held a higher educational degree^(29)^. In the current study, 82 % of the 4^th^ graders and 71 % of the 8^th^ graders had a least on parent/guardian with higher education, indicating that this sample is a selection of children and adolescents from highly educated families. As it is well known that lower socio-economic status is associated with a more unhealthy diet^(38)^, this may also affect the generalizability of the study. The data on body weight and height were self-reported, which is a weakness to the study as weight is often underestimated whereas height is overestimated^(39)^. In the present study, energy intake among the participants was probably underreported as previous results in the same study population showed that more than one-third of the children and adolescents were identified as under-reporters of energy^(16)^. As unhealthy foods most typically are underreported due to social desirability bias^(40)^, this might indicate that children and adolescents in Norway consume even more discretionary foods and drinks than was seen in the results from the present study.

Lastly, the data from the present study were collected in 2015, and changes may have occurred in the last 8 years.

## Conclusion

This study found that Norwegian children and adolescents consume about 20 % of their total energy intake from discretionary foods and drinks. Discretionary foods and drinks contributed to about two-thirds of the children and adolescents’ intake of added sugar, and limited amounts of dietary fibre, vitamins and minerals. Supper was the meal that contributed the most to the children’s and adolescents’ intake of discretionary foods. The quartile which had the lowest proportion of their energy intake from discretionary foods seemed to have a higher intake of whole grains, and fish and seafood in their diet compared to the upper quartile, suggesting healthier and more sustainable food habits.
